# Type I collagen deposition via osteoinduction ameliorates YAP/TAZ activity in 3D floating culture clumps of mesenchymal stem cell/extracellular matrix complexes

**DOI:** 10.1186/s13287-018-1085-9

**Published:** 2018-12-07

**Authors:** Nao Komatsu, Mikihito Kajiya, Souta Motoike, Manabu Takewaki, Susumu Horikoshi, Tomoyuki Iwata, Kazuhisa Ouhara, Katsuhiro Takeda, Shinji Matsuda, Tsuyoshi Fujita, Hidemi Kurihara

**Affiliations:** Department of Periodontal Medicine, Applied Life Sciences, Institute of Biomedical & Health Sciences, Graduate School of Biomedical & Health Sciences, Hiroshima University, Minami-ku, Kasumi 1-2-3, Hiroshima, Hiroshima 734-8553 Japan

**Keywords:** Mechanotransduction, 3D culture, YAP/TAZ, F-actin, C-MSCs

## Abstract

**Background:**

Three-dimensional (3D) floating culture clumps of mesenchymal stem cell (MSC)/extracellular matrix (ECM) complexes (C-MSCs) consist of cells and self-produced ECM. Previous studies have demonstrated that C-MSCs can be transplanted into bony lesions without an artificial scaffold to induce bone regeneration. Moreover, osteoinductive medium (OIM)-treated C-MSCs (OIM-C-MSCs) have shown rapid and increased new bone formation in vivo. To apply OIM-C-MSCs for novel bone regenerative cell therapy, their cellular properties at the molecular level must be elucidated. The transcriptional co-activators yes-associated protein/transcriptional co-activator with PDZ-binding motif (YAP/TAZ) have been recognized as key players in the mechanotransduction cascade, controlling cell lineage commitment in MSCs. It is plausible that 3D C-MSCs/OIM-C-MSCs cultured in floating conditions could provide distinct microenvironments compared to conventional 2D culture systems and thereby induce unique mechanotransduction cascades. Therefore, this study investigated the YAP/TAZ activity in 3D-cultured C-MSCs/OIM-C-MSCs in floating conditions.

**Methods:**

Human bone marrow-derived MSCs were cultured in growth medium supplemented with ascorbic acid. To obtain C-MSCs, confluent cells that had formed on the cellular sheet were scratched using a micropipette tip and were then torn off. The sheet was rolled to make round clumps of cells. Then, YAP/TAZ activity, filamentous actin (F-actin) integrity, collagen type I (COL1) production, and the differentiation potency in 3D floating culture C-MSCs/OIM-C-MSCs were analyzed.

**Results:**

C-MSCs cultured in floating conditions lost their actin cytoskeleton to downregulate YAP/TAZ activity, which directed cells to undergo adipogenesis/chondrogenesis. OIM treatment induced abundant COL1 deposition, which facilitated Intβ1-dependent actin fiber formation and YAP/TAZ activity to elevate the expression levels of osteogenic master transcriptional factor runt-related transcription factor 2 (RUNX2) mRNA in C-MSCs. Importantly, elevation of YAP/TAZ activity via OIM was associated with COL1 deposition and F-actin integrity, suggesting a positive feedback loop in OIM-C-MSCs.

**Conclusion:**

These findings suggest that OIM-C-MSCs, which form a unique microenvironment that maintains high YAP/TAZ activity, can serve as better candidates for bone regenerative cell therapy than C-MSCs.

**Electronic supplementary material:**

The online version of this article (10.1186/s13287-018-1085-9) contains supplementary material, which is available to authorized users.

## Background

Mesenchymal stem cells (MSCs), which possess self-renewing properties, multipotencey, and immunomoduratory potential, are currently the most extensively studied cells for tissue regenerative cell therapy, mainly due to their relative ease of isolation compared to other multipotent cells, such as embryonic stem cells and induced pluripotent stem cells [1]. For the tissue regenerative cell therapy, one of the common approaches is to seed MSCs on scaffold or microcarrier. However, despite recent advances, usage of the artificial scaffolds still harbors several limitations, including biodegradability and host unfavorable inflammatory response [2]. Indeed, our previous study demonstrated that MSC transplantation with osteointegrative biomaterial, β-tricalcium phosphate, induced alveolar bone regeneration in dog class III furcation defect model, though ankylosis, which is an abnormal adhesion of bone and periodontal ligament, was observed [3]. Accordingly, to solve this problem, we recently established clumps of MSC/extracellular matrix (ECM) complexes (C-MSCs), which consist of cells and self-produced ECM [4–7]. Three-dimensional (3D)-cultured C-MSCs can be grafted into bony lesions without any artificial scaffolds to induce bone regeneration. In addition, osteoinductive medium (OIM)-treated C-MSCs (OIM-C-MSCs) show rapid and increased new bone formation [4, 6]. These previous findings have suggested that OIM-C-MSCs may provide a promising bone regenerative therapy that utilizes cells while avoiding problems associated with the biocompatibility/biodegradability of artificial scaffolds. To develop such a novel cell therapy based on scientific evidence, it is necessary to distinguish the biological capacities of C-MSCs and OIM-C-MSCs at the molecular level.

Compared to traditional cell monolayers cultured on two-dimensional (2D) plastic or glass plates, 3D cultures of C-MSCs could provide markedly different and unique microenvironments for cells. For instance, 2D collagen-coated plastic plates provide a high-stiffness substrate, a continuous layer of ECM, and sufficient space for cells to spread [8, 9]. Conversely, 3D culture systems using various types of gels exhibit decreased stiffness, discrete matrix fibrils, and limited space, restricting cell shapes to a rounded form [8, 9]. Importantly, using mechanotransduction systems, such as those including integrins and cytoskeletal tension, cells can translate physical and mechanical cues into biochemical signals to regulate cell growth and differentiation. Indeed, cell density, cell shape, and matrix elasticity have been shown to direct MSC lineage commitment via cytoskeletal mechanics [10–12]. Accordingly, it is conceivable that 3D C-MSCs cultured in floating conditions could provide distinct microenvironments compared to conventional 2D culture systems, thereby resulting in the induction of a unique mechanotransduction pathway that regulates cell lineage specification and function.

The transcriptional co-activators yes-associated protein (YAP) and transcriptional co-activator with PDZ-binding motif (TAZ), originally identified as downstream targets of the Hippo signaling pathway [13–15], have recently been recognized as key factors in the mechanotransduction cascade [16–18]. Cellular microenvironments that tend to produce high intracellular resistive forces via actin fibers, including an extensive adhesion area, low cell density, stiff ECM, or fluid shear stress, facilitate YAP/TAZ localization to the nucleus to activate their transcriptional activities. In contrast, a narrow adhesion area, high cell density, soft ECM, and disturbed fluid flow produce a low cytoskeletal contractile force by disrupting the integrity of actin fibers, which causes YAP/TAZ cytoplasmic retention and proteasomal degradation [15, 19, 20]. Intriguingly, DuPont et al. reported that MSCs, by sensing the mechanical cues that facilitate the integrity of actin (e.g., stiff ECM), were induced to form osteoblasts via high YAP/TAZ activity, whereas cells cultured on soft ECM or treated with an acto-myosin inhibitor showed low YAP/TAZ activity, consistent with adipogenesis [21].

Based on these accumulating lines of evidence, we hypothesized that the microenvironment surrounding 3D floating cultured C-MSCs, which should be different from traditional 2D culture, may cause unique mechanotransduction through cytoskeletal mechanics and YAP/TAZ activity, which, in turn, affects the direction of cells’ lineage commitment. Accordingly, the present study investigated (1) filamentous actin (F-actin) fiber integrity and YAP/TAZ activity in 3D floating culture C-MSCs and (2) the effects of OIM on the mechanotransduction cascade that underlies the molecular mechanism of osteogenic induction in C-MSCs.

## Materials and methods

### C-MSC preparation and culture

**Fig. 1:**
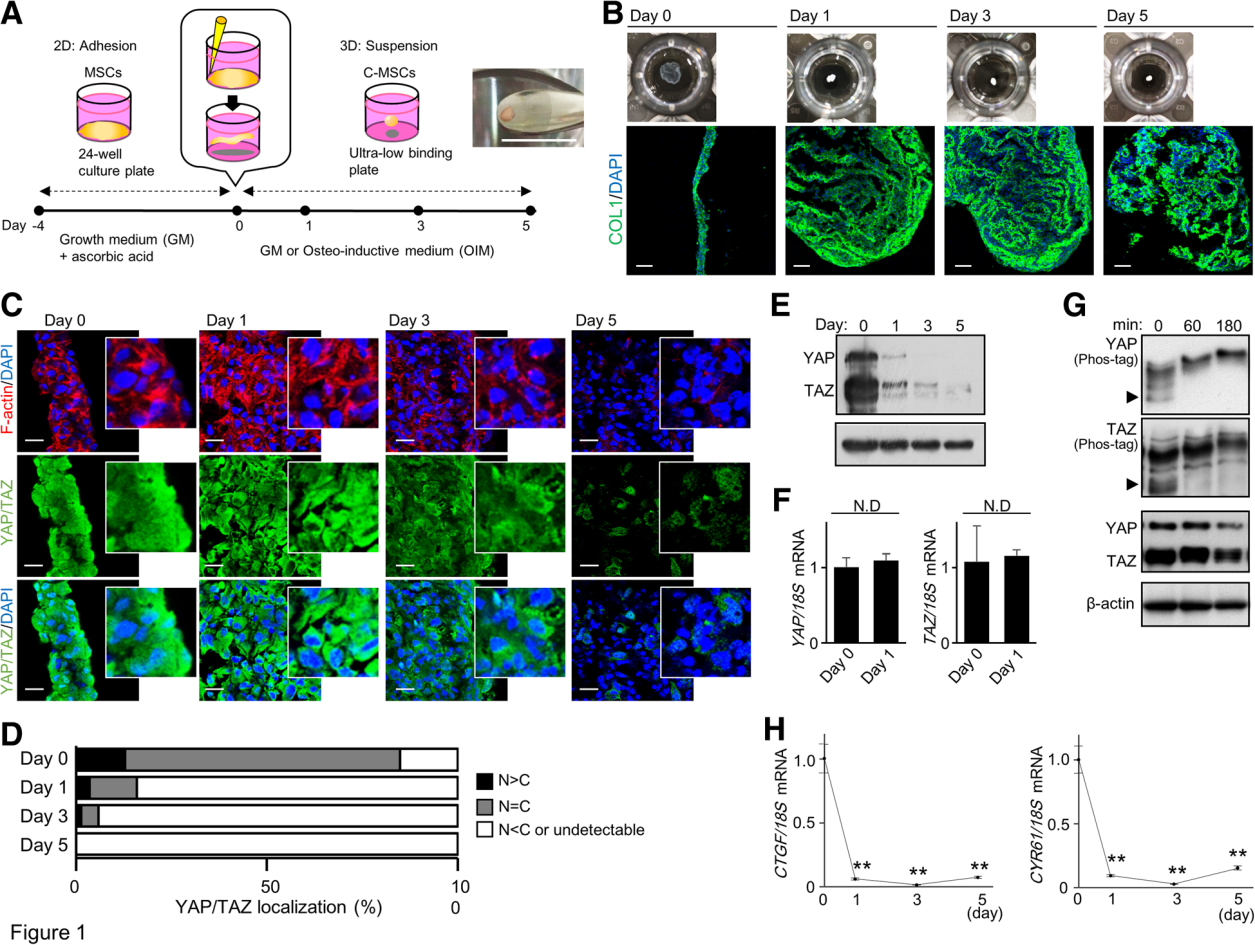
YAP and TAZ are downregulated in 3D-cultured C-MSCs. **a** Schematic image of C-MSC culture. Bar = 5 mm. **b**–**g** C-MSCs were generated and maintained in growth medium (GM) for the indicated culture periods. **b** Upper panels indicate macroscopic images of C-MSCs. Lower panels show confocal immunofluorescence images of COL1 and nuclei in C-MSCs. Bar = 100 μm. **c** Confocal immunofluorescence images of YAP/TAZ (green), F-actin (red), and nuclei (blue) in C-MSCs. Bar = 20 μm. White boxes show enlarged images. **d** The graph summarizes the distribution of YAP/TAZ patterns. The patterns were classified as mainly nuclear (*N* > *C*), diffuse (*N* = *C*) and mainly cytoplasmic or undetectable (*N* < *C* or undetectable). **e** Immunoblotting for YAP/TAZ in C-MSCs. **f**, **h** Real-time PCR for YAP and TAZ (**f**) or YAP/TAZ target genes (**h**) in C-MSCs. Data were normalized to the values on day 0. Values represent means ± S.D. of three cultures (**p < 0.01). **g** Phos-tag-SDS-PAGE was used to show the phosphorylation levels of YAP/TAZ. Arrowheads indicate the position of unphosphorylated YAP/TAZ. Levels of total YAP/TAZ were analyzed by immunoblotting. All graphs and images are representative of three independent experiments

Human bone marrow MSCs were purchased from Lonza (Lonza, Basel, Switzerland) or were provided by RIKEN BioResource Center (Ibaragi, Tsukuba, Japan). The cells were cultured in DMEM (Sigma, Steinheim, Germany) supplemented with 10% fetal bovine serum (FBS) (HyClone, Logan, UT), 100 U/mL penicillin (Sigma), and 100 μg/mL streptomycin (Sigma). Then, C-MSCs were generated as described previously [5]. Briefly, MSCs were seeded at a density of 2.0 × 10^5^ cells/well in 24-well plates (Corning, Corning, NY) and cultured with high-glucose DMEM (Sigma) supplemented with 10% fetal FBS, 100 U/mL penicillin, and 100 μg/mL streptomycin (growth medium (GM)) in the presence of 50 μg/mL L-ascorbic acid (Sigma) for 4 days. To obtain C-MSCs, we took confluent cells that had formed a cellular sheet, consisting of self-produced ECM, and detached them from the bottom of the plate by scratching the edge with a micropipette tip. The floating MSC/ECM complexes were transferred to a 24-well ultra-low-binding plate (Corning) and cultured with GM or OIM (GM supplemented with 100 nM dexamethasone (Sigma), 50 μg/mL L-ascorbic acid, and 10 mM β-glycerophosphate (Sigma)) in the presence or absence of Y27632 (50 μM, Chemdea, Ridgewood, NJ) or blebbistatin (50 μM, Cayman Chemical, Ann Arbor, MI) for 5 days. After 1 day of incubation, the cellular sheets rolled up, producing round clumps of cells, and 0.8–1.2-mm-diameter C-MSCs were obtained (Fig. 1a). All experiments were repeated at least three times independently.

### Transfection

siRNAs targeting YAP, TAZ, or negative control siRNA [21] were chemically synthesized and provided by FASMAC (FASMAC, Kanagawa, Japan). The sequences of these siRNAs are listed in Additional file 1: Table S1. Validated integrin β1 (Intβ1) siRNA was obtained from Invitrogen (#HSS105561; Invitrogen, Carlsbad, CA). Two days before the production of C-MSCs (before scratching), siRNAs were transfected into cells using Lipofectamine RNAi-MAX (Invitrogen) according to the manufacturer’s instructions. Then, the cell sheets were detached and transferred to a 24-well ultra-low-binding plate to generate C-MSCs as described above. The knockdown effects of each siRNA were assessed (Additional file 2: Figure S1A-S1C). All experiments were repeated at least three times independently.

The expression plasmid for HA-TAZS89A was obtained from Addgene (Addgene plasmid 32840; Addgene, Cambridge, MA). An HA expression plasmid was employed as a control (#635688; Clontech, Palo Alto, CA). DNA transfections were performed with ECM 830 (BTX, Holliston, MA). Briefly, after detaching the MSC/ECM complexes from the culture plate, the cell sheet was suspended in 100 μL of Opti-MEM (Invitrogen) with 4 μg of each DNA plasmid and placed into 4-mm-gap cuvettes. Transfections were conducted at 300 V with a 1 ms pulse length and 10 pulses over 5 s. After the pulses, the cell sheets were transferred to a 24-well ultra-low-binding plate to generate C-MSCs, as described above. The effects of each plasmid transfection were assessed (Additional file 2: Figure S1D and S1E). All experiments were repeated at least three times independently.

### Induction of C-MSCs

For osteogenic, adipogenic, or chondrogenic induction, the cell sheets were detached from the culture plates and maintained in OIM, adipogenic induction medium (AIM; high-glucose DMEM supplemented with 10% FBS, 0.5 mM isobutylmethylxanthine (Nacalai Tesque, Kyoto, Japan), 0.1 mM indomethacin (Sigma), 1 μM dexamethasone (Sigma), and 10 μg/mL insulin (Sigma)), chondrogenic induction medium (CIM; high-glucose DMEM supplemented with ITS™+ Premix (Corning), 1 mM sodium pyruvate (Sigma), 0.35 mM proline (Sigma), 10 ng/mL transforming growth factor-β3 (Aviscera Bioscience, Santa Clara, CA), 100 nM dexamethasone, and 0.17 mM L-ascorbic acid), or dual induction medium (dual; equal volumes of OIM and AIM) [22] for 5 days. All experiments were repeated at least three times independently.

### Immunofluorescence analysis

C-MSCs were fixed with 4% paraformaldehyde in PBS. The fixed samples were embedded in Tissue-Tek OCT compound (Sakura, Torrance, CA), and 20-μm-thick sections were cut using a cryostat. The sections were washed with PBS and then treated with 5% BSA and 0.1% TWEEN 20 in PBS to block nonspecific staining. These sections were incubated with a rabbit anti-human YAP/TAZ IgG antibody (D24E4; 1:1000; Cell Signaling, Beverly, MA) or rabbit anti-human type I collagen polyclonal IgG (1:200; Novus Biologicals, Littleton, CO) at 4 °C overnight. After being washed 3 times with PBS, samples were incubated for 2 h with an Alexa Fluor 488® goat anti-rabbit IgG antibody (1:100; Invitrogen) and Alexa Fluor 594® phalloidin (1:50; Invitrogen) at room temperature. Then, the nuclei were counterstained with DAPI (5 μg/mL; Invitrogen). After washing the samples with PBS, we detected the fluorescence signals using a Zeiss LSM 5 PASCAL laser scanning confocal microscope (Carl Zeiss Microimaging, Inc., Oberkochen, Germany). To assess YAP/TAZ distribution patterns, we quantified cells in three random fields of view (*n* = 150~200) as previously described [23].

### Immunoblotting

The C-MSCs were lysed in buffer containing 25 mM Tris-HCl (pH 7.4), 150 mM NaCl, 5 mM EDTA (pH 8.0), 0.1% SDS, 1% NP-40, 10% glycerol, and 1% (*v*/v) Triton X-100 [24]. The cell lysates were subjected to ultrasonic treatment for 8 s on ice. Proteins in the lysates were subjected to SDS-PAGE and transferred to a nitrocellulose membrane (Bio-Rad Laboratories, Hercules, CA). The membranes were blocked for 1 h with 5% skim milk and then immunoblotted using rabbit anti-YAP/TAZ IgG (D24E4; 1:1000; Cell Signaling), rabbit anti-YAP IgG (D8H1X; 1:1000; Cell Signaling), rabbit anti-TAZ IgG (V386; 1:1000; Cell Signaling), or mouse anti-β-actin monoclonal antibodies (AC-15; 1:10000; Sigma). The membranes were developed using ECL Plus Western blotting detection reagents (GE Healthcare, Little Chalfont, UK).

For detection of phosphorylated YAP and TAZ, cell lysates were subjected to SDS-PAGE containing a phosphorylation tag (Phos-Tag™, Wako, Osaka, Japan) that specifically traps phosphorylated proteins in the gel. After immunoblotting with anti-YAP or anti-TAZ IgG antibodies, phosphorylated proteins were detected as slower-migrating bands compared to the corresponding unphosphorylated proteins.

### Real-time polymerase chain reaction

Total RNA from cultured C-MSCs was extracted using RNA-iso® (Takara, Otsu, Japan) and quantified by spectrometry at 260 and 280 nm. RNA samples (500 ng) were reverse-transcribed into complementary DNA using ReverTra Ace (Toyobo, Osaka, Japan). Then, real-time PCR was performed using a LightCycler and SYBR green (Roche Applied Science, Mannheim, Germany) to determine the relative mRNA expression of *collagen type I* (*COL1*), *connective tissue growth factor* (*CTGF*), *cysteine-rich angiogenic inducer 61* (*CYR61*), *YAP*, *TAZ*, *runt-related transcription factor 2* (*RUNX2*), *osteocalcin* (*OCN*), *peroxisome proliferator-activated receptor γ* (*PPARγ*), *activating protein 2* (*AP2*), *sex determining region Y-box 9* (*SOX9*), and *Aggrecan*. The PCR thermal profile consisted of an initial 10 min at 95 °C, followed by 40 cycles of 95 °C for 15 s and 60 °C for 1 min. Fold changes of gene of interest were calculated with ∆∆Ct method using ribosomal protein *18S* as reference control. The sequences of the primers used in this study are listed in Additional file 1: Table S2.

### Statistical analysis

Experiments were repeated three or four times, and the results are expressed as the means ± SD. Differences between parameters were tested for statistical significance using unpaired Student’s *t* tests or ANOVA. Values of *p* < 0.05 or *p* < 0.01 were considered significant.

## Results

### YAP/TAZ activity is downregulated in 3D-cultured C-MSCs

For the generation of 3D-cultured C-MSCs, confluent MSCs treated with ascorbic acid for 4 days were scratched using the tip of a micropipette (Fig. 1a). The MSC/ECM complexes were detached from the culture plate and exhibited a sheet-like morphology (Fig. 1b). After 1 day, this cellular sheet rolled up to form round cell clumps, termed C-MSCs. Immunofluorescence analysis demonstrated that C-MSCs were formed from COL1 produced by the MSCs during the culture period (Fig. 1b). During the C-MSC culture process, F-actin and YAP/TAZ expression patterns were assessed by confocal microscopy. F-actin was thick and abundant at the beginning of C-MSC culture; however, it became thinner and degraded over time, suggesting that F-actin depolymerization occurred in a time-dependent manner (Fig. 1c). In accordance with F-actin depolymerization, YAP/TAZ translocated to the cytoplasm (Fig. 1c, d), and their fluorescence signals were difficult to detect in cultured C-MSCs on day 5 (Fig. 1c). In addition, Western blotting demonstrated that the YAP/TAZ protein levels decreased dramatically on day 1, and this reduction was time-dependent (Fig. 1e). In contrast, their mRNA expression levels were unchanged (Fig. 1f), suggesting that the downregulation of YAP/TAZ in C-MSCs may not be due to transcriptional reduction but instead to proteasomal degradation. It is widely known that phosphorylation of YAP/TAZ leads to their cytoplasmic retention and degradation, which results in the inhibition of YAP/TAZ transcriptional activity. Therefore, next, we measured the phosphorylation levels of YAP/TAZ before their degradation. Notably, YAP/TAZ mobility shifts in a phos-tag gel, suggesting their phosphorylation, were induced in a time-dependent manner after 60 min of detachment (Fig. 1g). Consistent with these findings, the mRNA expression levels of the YAP/TAZ transcriptional target genes *CTGF* and *CYR61* were also markedly decreased on day 1, and the levels remained reduced until the end of the culture period (Fig. 1h). In contrast, MSCs maintained on 2D plastic culture plates kept abundant F-actin fiber structure and demonstrated only slight reductions in YAP/TAZ activity on day 5 (Additional file 2: Figure S2). These findings clearly indicated that YAP/TAZ activity is downregulated in association with actin degradation in 3D floating cultured C-MSCs.

### 3D-cultured C-MSCs can be directed towards adipogenesis and chondrogenesis, but not osteogenesis, because of reduced YAP/TAZ activity

**Fig. 2:**
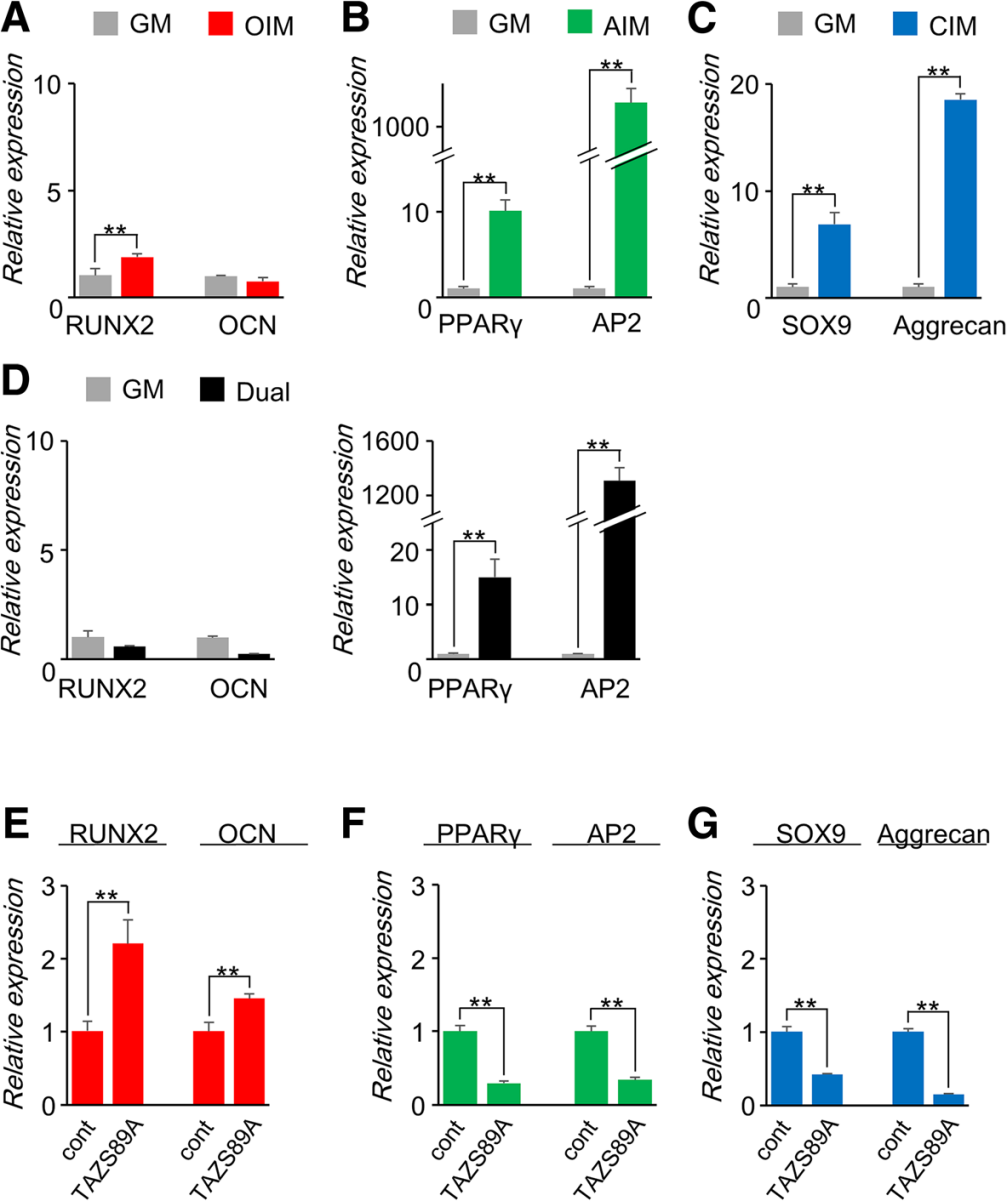
Three-dimensional floating culture C-MSCs can be directed towards adipogenesis/chondrogenesis but not osteogenesis due to reduced YAP/TAZ activity. **a**–**d** C-MSCs were cultured in **a** OIM, **b** AIM, **c** CIM, or **d** dual medium for 5 days. Differentiation marker gene expression levels were analyzed by real-time PCR. Data were normalized to the values of C-MSCs maintained in GM. **e**–**g** C-MSCs transfected with a constitutively active TAZ mutant (TAZS89A) or control plasmid were maintained in **e** OIM, **f** AIM, or **g** CIM for 5 days. Differentiation marker gene expression levels were analyzed by real-time PCR. Data were normalized to the values of C-MSCs transfected with control vector (cont). Values represent means ± S.D. of three cultures (**p < 0.01). All graphs are representative of three independent experiments

Because it is well established that downregulation of YAP/TAZ is responsible for adipogenesis/chondrogenesis but not osteogenesis in MSCs [21, 22], we hypothesized that C-MSCs, which showed decreased YAP/TAZ activity, could be directed towards adipo-/chondrogenesis. To test this hypothesis, various induction patterns were examined. OIM treatment caused a twofold increase in *RUNX2* mRNA expression, although *OCN* mRNA levels were not affected by OIM (Fig. 2a). Furthermore, AIM or CIM dramatically increased *PPARγ* and *AP2* or *Sox9* and *Aggrecan* mRNA expression levels in C-MSCs, respectively (Fig. 2b, c). Dual induction, consisting of equal volumes of OIM and AIM, markedly elevated adipogenic marker genes but not osteogenic genes (Fig. 2d). Importantly, overexpression of a constitutively active form of TAZ (TAZS89A) significantly increased both *RUNX2* and *OCN* mRNA expression levels in C-MSCs cultured with OIM (Fig. 2e). However, TAZS89A-transduced C-MSCs demonstrated decreased levels of adipogenic and chondrogenic marker gene expression with AIM and CIM treatment, respectively (Fig. 2f, g). These results indicated that 3D floating cultured C-MSCs are susceptible to adipo-/chondrogenic induction because of low YAP/TAZ activity.

However, it is possible that our primary MSCs are preferentially directed into adipo-/chondrogenesis instead of osteogenesis. To examine this possibility, we investigated the effects of each induction medium on cells cultured on 2D plastic culture plates at low cell densities were investigated. Consistent with previous studies [21], subconfluent MSCs on 2D culture plates showed high YAP/TAZ activity (Additional file 2: Figure S3A and S3B). Importantly, 2D-cultured cells demonstrated remarkable increases in osteogenesis but only slight or insufficient adipogenesis and chondrogenesis (Additional file 2: Figure S3C-S3F). In addition, knockdown of YAP/TAZ by siRNA transfection reduced osteogenesis in 2D-cultured MSCs, complementing increases in adipogenesis and chondrogenesis (Additional file 2: Figure S3H-S3J). These findings indicate that our cells possess osteogenic properties in culture conditions with increased YAP/TAZ activity. Therefore, the adipo-/chondrogenic properties of 3D-cultured C-MSCs are due to reduced YAP/TAZ activity.

### OIM facilitates F-actin formation and YAP/TAZ activity in C-MSCs

**Fig. 3:**
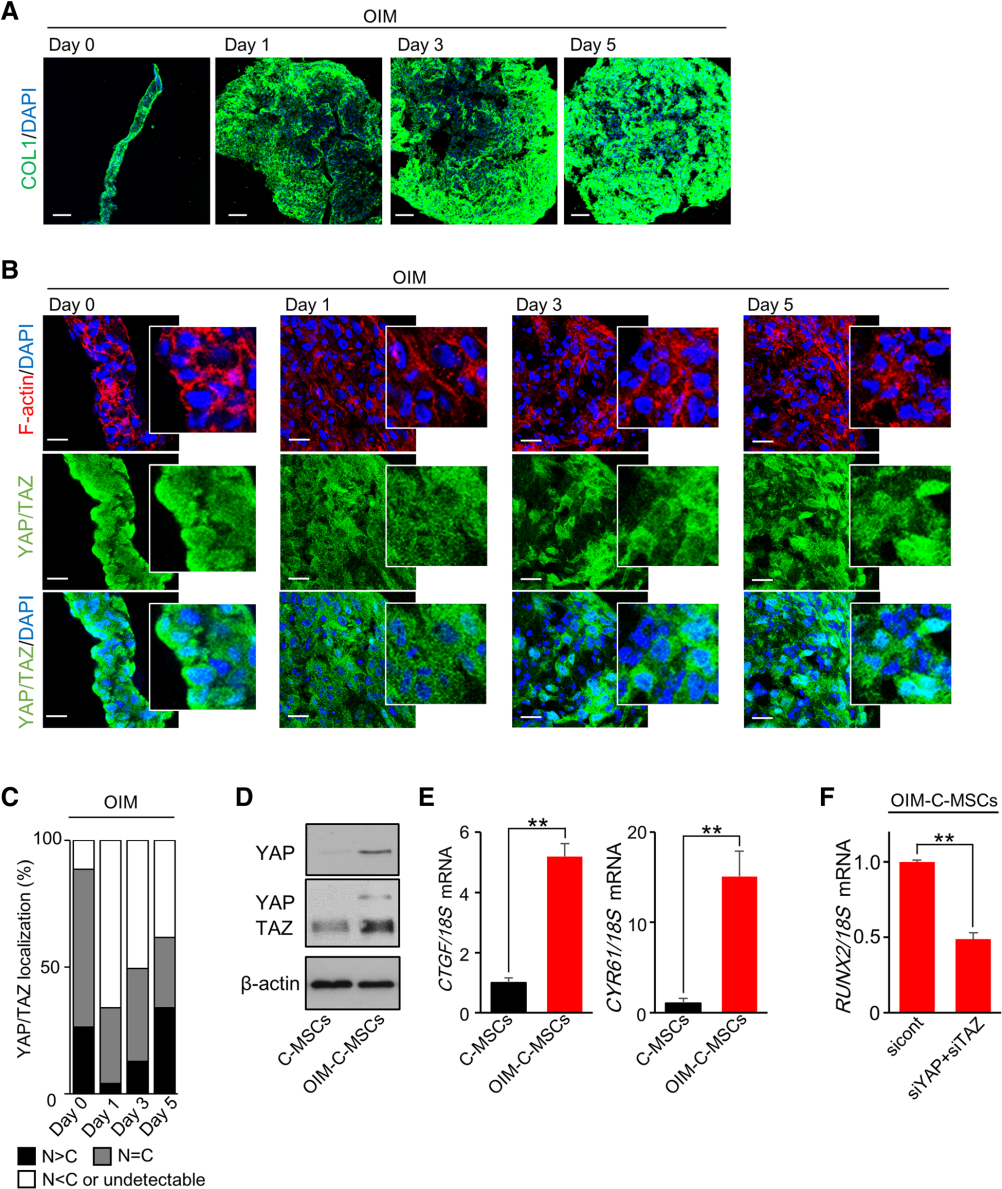
Osteoinductive medium facilitates F-actin formation and YAP/TAZ activity in C-MSCs. **a**, **b** C-MSCs were cultured in OIM for the indicated culture period. **a** Confocal immunofluorescence images of COL1 and nuclei. Bar = 100 μm. **b** Confocal immunofluorescence images of YAP/TAZ (green), F-actin (red), and nuclei (blue) in C-MSCs. Bar = 20 μm. White boxes show enlarged images. **c** The graph summarizes the distribution of YAP/TAZ localization patterns. The patterns were classified as mainly nuclear (*N* > *C*), diffuse (*N* = *C*), or mainly cytoplasmic or undetectable (*N* < *C* or undetectable). **d**–**f** C-MSCs: C-MSCs cultured in GM for 5 days. OIM-C-MSCs: C-MSCs cultured in OIM for 5 days. **d** Immunoblotting for YAP/TAZ. A rabbit anti-YAP/TAZ (D24E4) mAb (Cell Signaling) was used to detect both the YAP and TAZ proteins (middle panel). To show YAP expression more clearly, we also used a rabbit anti-YAP (D8H1X) mAb (upper panel). **e** Real-time PCR of YAP/TAZ target genes. Data were normalized to the values of C-MSCs. Values represent means ± S.D. of three cultures (**p < 0.01). **f** C-MSCs transfected with negative control or YAP and TAZ siRNAs were cultured in OIM for 5 days. *RUNX2* expression levels were analyzed by real-time PCR. Data were normalized to the values of OIM-C-MSCs transfected with negative control siRNA. Values represent means ± S.D. of three cultures (**p < 0.01). All graphs and images are representative of three independent experiments

Previously, we reported that OIM-C-MSCs (C-MSCs cultured with OIM for 5 days) exert more effective bone regenerative properties in vivo than C-MSCs [4, 6]. Moreover, OIM-C-MSCs showed slightly but significantly increased *RUNX2* mRNA expression, as shown in Fig. 2a, indicating that the biological characteristics of OIM-C-MSCs could be more osteogenic than those of C-MSCs. Importantly, because it is widely accepted that high YAP/TAZ activity is responsible for osteogenesis in MSCs [21, 25], we next hypothesized that OIM treatment ameliorates YAP/TAZ activity in 3D floating cultured C-MSCs to induce osteogenesis. First, we determined that OIM treatment increased COL1 expression, which induces C-MSC morphology in a time-dependent manner (Fig. 3a). Importantly, unlike the YAP/TAZ and F-actin expression patterns in the C-MSCs described above (Fig. 1), C-MSCs treated with OIM maintained thick, abundant F-actin fibers, and YAP/TAZ expression for 5 days (Fig. 3b). In addition, OIM treatment restored the level of nuclear YAP/TAZ in C-MSCs, which was decreased after detachment for 1 day (Fig. 3b, c). Compared to C-MSCs, OIM-C-MSCs showed higher YAP/TAZ levels (Fig. 3d) and significantly increased YAP/TAZ transcriptional activity (Fig. 3e). Therefore, YAP/TAZ siRNAs were used to determine whether this elevated YAP/TAZ activity was associated with *RUNX2* mRNA expression in OIM-C-MSCs. Co-transfection of YAP/TAZ siRNAs significantly decreased *RUNX2* mRNA expression levels in OIM-C-MSCs (Fig. 3f). These findings indicated that OIM treatment facilitates YAP/TAZ activity to induce *RUNX2* mRNA expression in 3D floating cultured C-MSCs.

### COL1 deposition caused by OIM facilitates a mechanotransduction cascade composed of Intβ1-rho-associated protein kinase (ROCK)-F-actin-YAP/TAZ signaling to induce osteogenesis in C-MSCs

**Fig. 4:**
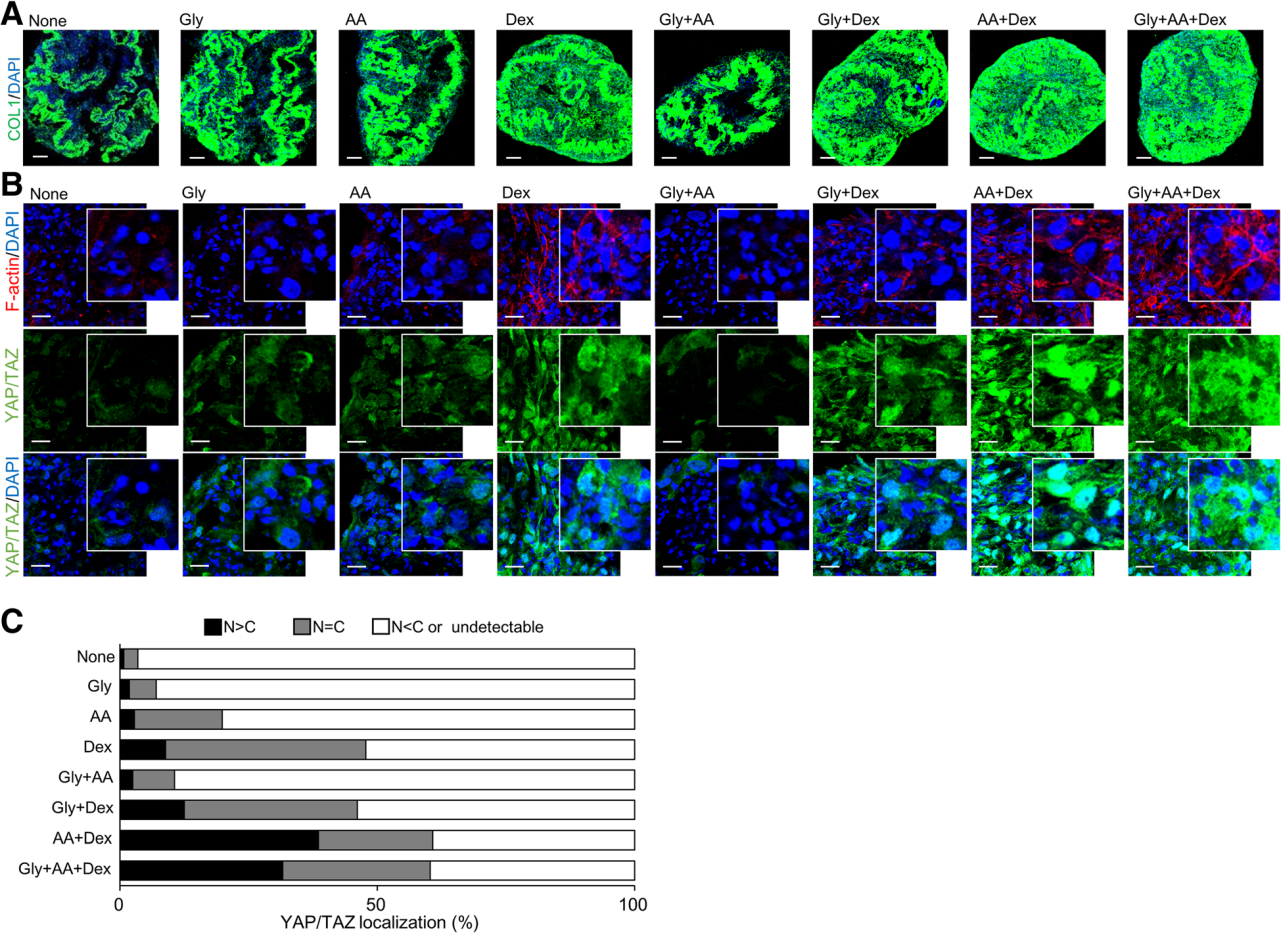
The effects of OIM components on COL1 production, F-actin integrity, and YAP/TAZ activity in C-MSCs. **a**–**c** C-MSCs were cultured with GM (none) in the presence of β-glycerophosphate (Gly), ascorbic acid (AA), and/or dexamethasone (Dex) (Gly+AA, Gly+Dex, AA+Dex, and Gly+AA+Dex (equivalent to OIM)) for 5 days. **a** Confocal immunofluorescence images of COL1 (green) and nuclei (blue) in C-MSCs. Bar = 100 μm. **b** Confocal immunofluorescence images of YAP/TAZ (green), F-actin (red), and nuclei (blue) in C-MSCs. Bar = 20 μm. **c** The graph summarizes the distribution of YAP/TAZ patterns. The patterns were classified as mainly nuclear (*N* > *C*), diffuse (*N* = *C*), and mainly cytoplasmic or undetectable (*N* < *C* or undetectable)

**Fig. 5:**
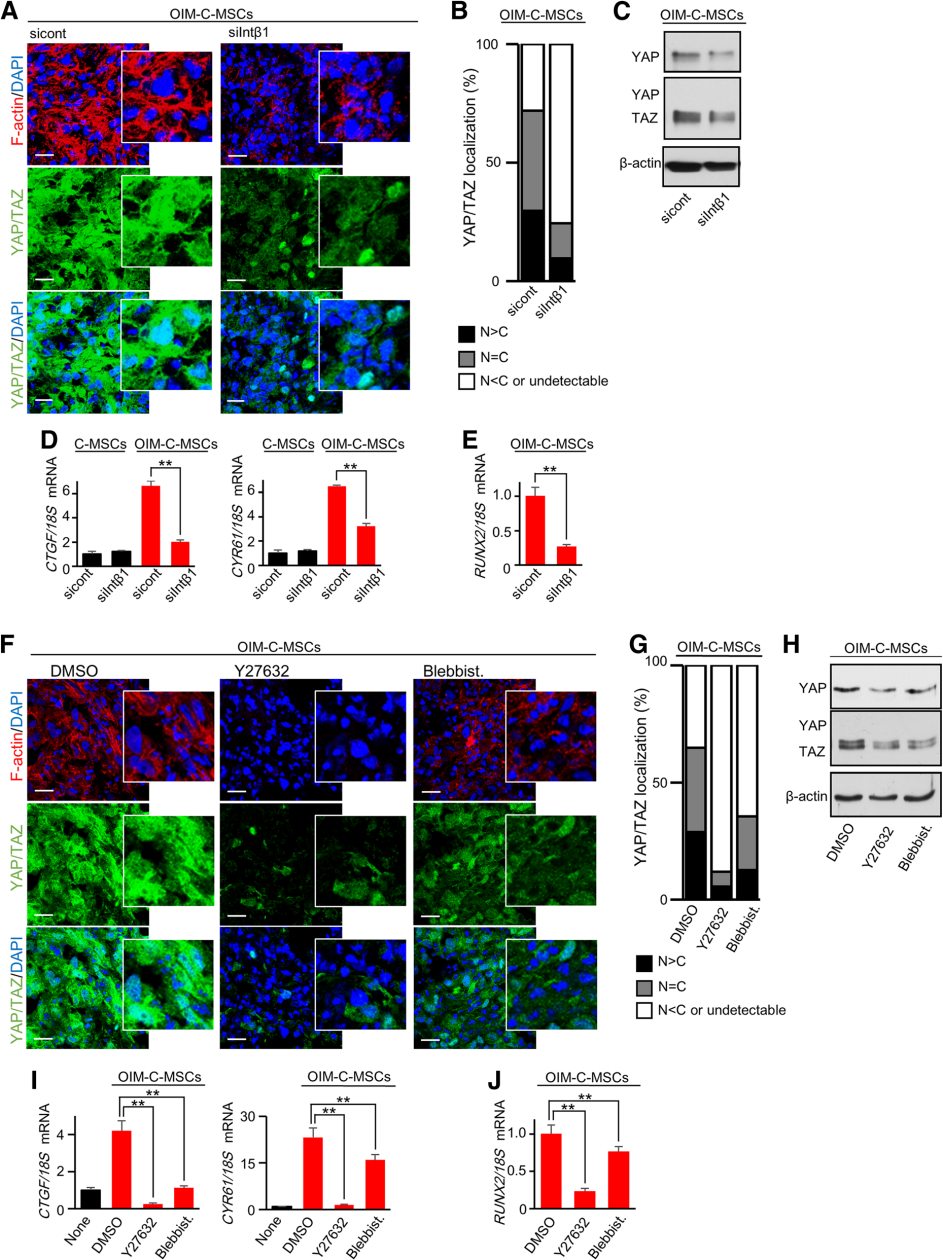
OIM-induced YAP/TAZ activity in C-MSCs is due to actin cytoskeletal tension caused by Intβ1-ROCK signaling. **a**–**j** C-MSCs: C-MSCs cultured with GM for 5 days. OIM-C-MSCs: C-MSCs cultured with OIM for 5 days. **a**–**e** C-MSCs transfected with negative control or Intβ1 siRNAs were cultured with GM or OIM for 5 days. **a** Confocal immunofluorescence images of YAP/TAZ (green), F-actin (red), and nuclei (blue) in OIM-C-MSCs. Bar = 20 μm. White boxes show enlarged images. **b** The graph summarizes the distribution of YAP/TAZ localization patterns. The patterns were classified as mainly nuclear (*N* > *C*), diffuse (*N* = *C*), and mainly cytoplasmic or undetectable (*N* < *C* or undetectable). **c** Immunoblotting for YAP/TAZ in OIM-C-MSCs. A rabbit anti-YAP/TAZ (D24E4) mAb (Cell Signaling) was used to detect both the YAP and TAZ proteins (middle panel). To show YAP expression more clearly, we also used a rabbit anti-YAP (D8H1X) mAb (upper panel). **d** Real-time PCR analysis of YAP/TAZ target genes. Data were normalized to the values of control siRNA-transfected C-MSCs. Values represent means ± S.D. of three cultures (**p < 0.01). **e** *RUNX2* expression levels in OIM-C-MSCs were analyzed by real-time PCR. Data were normalized to the values of OIM-C-MSCs transfected with negative control siRNA. Values represent means ± S.D. of three cultures (**p < 0.01). All graphs and images are representative of three independent experiments. **f**–**j** C-MSCs or OIM-C-MSCs were cultured with or without a ROCK inhibitor (Y27632, 50 μM), the non-muscle myosin inhibitor blebbistatin (Blebbist., 50 μM), or an appropriate concentration of DMSO for 5 days. **f** Confocal immunofluorescence images of YAP/TAZ (green), F-actin (red), and nuclei (blue) in OIM-C-MSCs. Bar = 20 μm. White boxes show enlarged images. **g** The graph summarizes the distribution of YAP/TAZ localization patterns. The patterns were classified as mainly nuclear (*N* > *C*), diffuse (*N* = *C*), and mainly cytoplasmic or undetectable (*N* < *C* or undetectable). **h** Immunoblotting for YAP/TAZ in OIM-C-MSCs. A rabbit anti-YAP/TAZ (D24E4) mAb (Cell Signaling) was used to detect both the YAP and TAZ proteins (middle panel). To show YAP expression more clearly, we also used a rabbit anti-YAP (D8H1X) mAb (upper panel). **i** Real-time PCR analysis of YAP/TAZ target genes. Data were normalized to the values of C-MSCs treated with DMSO. Values represent means ± S.D. of three cultures (**p < 0.01). **j** *RUNX2* expression levels in OIM-C-MSCs were analyzed by real-time PCR. Data were normalized to the values of OIM-C-MSCs treated with DMSO. Values represent means ± S.D. of three cultures (**p < 0.01). All graphs and images are representative of three independent experiments

To clarify how OIM elevates YAP/TAZ activity in C-MSCs, we investigated the effect of each component in OIM, including β-glycerophosphate (Gly), ascorbic acid (AA), and dexamethasone (Dex), on COL1 production, F-actin expression, and YAP/TAZ localization in C-MSCs. Immunofluorescence staining demonstrated that Dex- or Gly+Dex-treated groups showed higher COL1 production, F-actin formation, and increased YAP/TAZ activity than the non-(GM only), Gly-, AA-, or Gly+AA-treated groups (Fig. 4a–c). Moreover, both AA+Dex and Gly+AA+Dex (OIM) treatment demonstrated the greatest amounts of COL1 deposition in C-MSCs (Fig. 4a). Consistent with this increased COL1 production, F-actin integrity and YAP/TAZ activity were also strongest in all tested groups (Fig. 4b, c). These findings suggested the possibility that COL1 deposition caused by OIM (mainly the Dex and AA components) plays a role in the upregulation of F-actin formation and YAP/TAZ activity. Accordingly, we investigated the effects of Intβ1 siRNA on YAP/TAZ activity in OIM-C-MSCs because Intβ1 links the ECM, including COL1, with the actin cytoskeleton, thereby transmitting signals from the ECM to the cytoplasm [26]. Notably, OIM-C-MSCs transfected with Intβ1 siRNA showed disruption of F-actin fibers (Fig. 5a). Importantly, knockdown of Intβ1 attenuated YAP/TAZ expression, and undegraded YAP/TAZ was localized mainly in the cytoplasm of OIM-C-MSCs (Fig. 5a, b). Western blotting demonstrated that Intβ1 siRNA treatment decreased YAP/TAZ expression levels in OIM-C-MSCs (Fig. 5c). Depletion of Intβ1 by siRNA abrogated *CTGF* and *CYR61* mRNA expression levels, suggesting the suppression of YAP/TAZ transcriptional activity (Fig. 5d). In addition, OIM-C-MSCs transfected with Intβ1 siRNA showed decreased expression levels of *RUNX2* mRNA (Fig. 5e). These findings indicated that OIM-induced COL1 could increase F-actin integrity and YAP/TAZ activity via Intβ1 signaling in 3D-cultured C-MSCs.

Because ROCK is well established as a downstream target of Intβ1, which facilitates actin polymerization to induce traction force, we next explored the relationship between OIM-induced YAP/TAZ activity and ROCK or the actin cytoskeletal tension using chemical inhibitors. Inhibition of ROCK activity by Y27632 treatment drastically diminished F-actin fibers, consistent with reductions in YAP/TAZ expression levels in OIM-C-MSCs (Fig. 5f). OIM-C-MSCs treated with blebbistatin showed disruption of actin fiber integrity, suggesting a reduction in cytoskeletal tension and degradation of YAP/TAZ (Fig. 5f). YAP/TAZ that had avoided degradation in both Y27632- and blebbistatin-treated groups were mainly localized in the cytoplasm (Fig. 5f, g). Western blotting also showed downregulation of YAP/TAZ expression in OIM-C-MSCs treated with Y27632 and blebbistatin (Fig. 5h). Real-time PCR demonstrated that the YAP/TAZ target genes *CTGF* and *CYR61* were reduced by treatment with these chemical inhibitors as well (Fig. 5i). In addition, *RUNX2* mRNA expression levels in OIM-C-MSCs were reduced by ROCK inhibition and F-actin disruption (Fig. 5j). These findings suggested that OIM-induced YAP/TAZ activity was dependent on F-actin fiber integrity regulation by ROCK in 3D-cultured C-MSCs.

### YAP/TAZ activity is associated with COL1 synthesis and F-actin formation, generating a positive feedback loop in OIM-C-MSCs

**Fig. 6:**
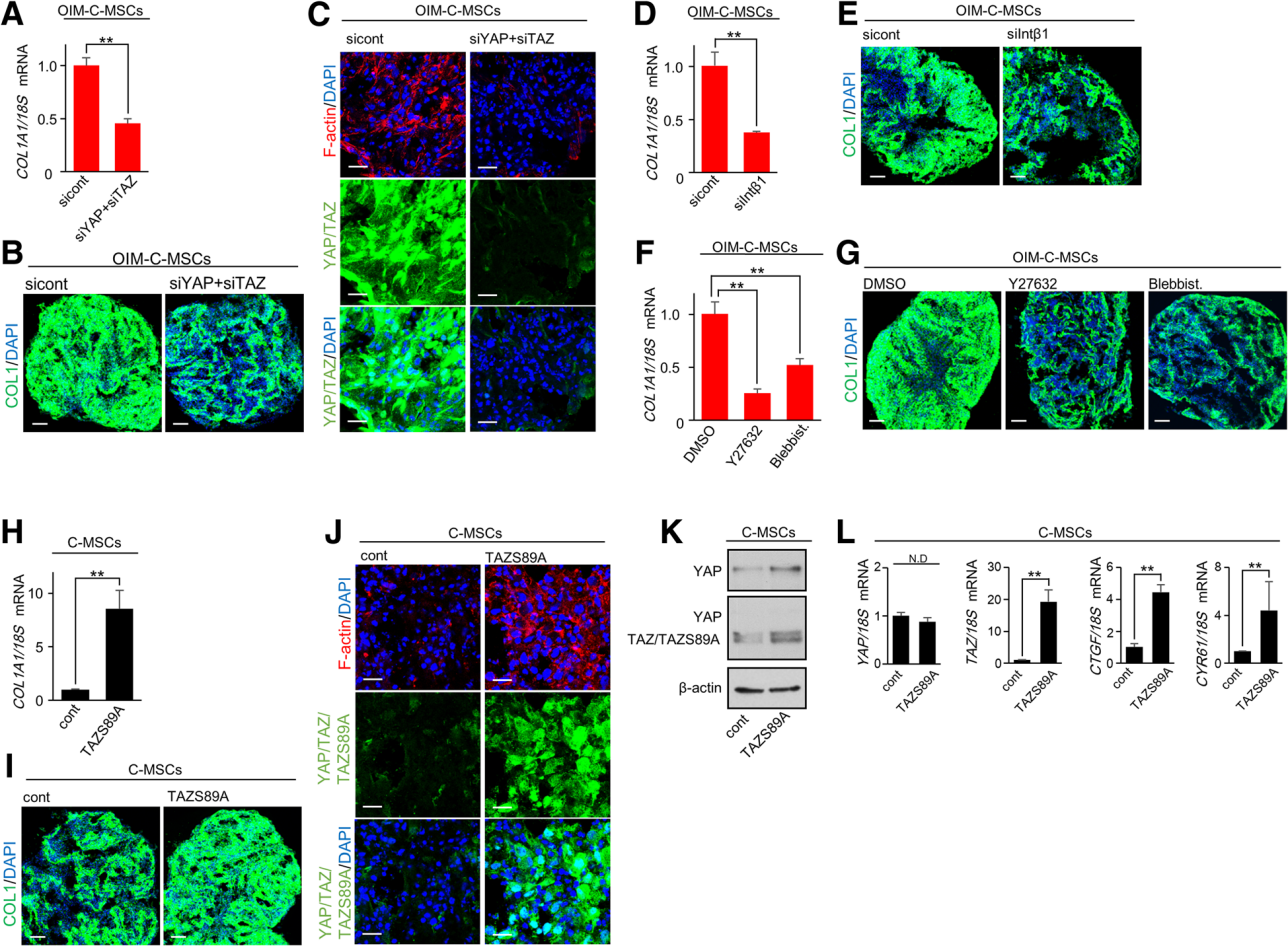
YAP/TAZ activity is associated with COL1 synthesis and F-actin formation, generating a positive feedback loop in OIM-C-MSCs. (**a**–**m**) C-MSCs: C-MSCs cultured with GM for 5 days. OIM-C-MSCs: OIM-C-MSCs cultured with OIM for 5 days. **a**–**e** C-MSCs transfected with negative control, YAP and TAZ, or Intβ1 siRNAs were cultured with OIM for 5 days. **a**, **d** *COL1A1* mRNA expression levels were analyzed by real-time PCR. Data were normalized to the values of OIM-C-MSCs transfected with negative control siRNA. Values represent means ± S.D. of three cultures (**p < 0.01). **b**, **e** Confocal immunofluorescence images show COL1 (green) and nuclei (blue) in OIM-C-MSCs. Bar = 100 μm. **c** Confocal immunofluorescence images of YAP/TAZ (green), F-actin (red), and nuclei (blue) in OIM-C-MSCs. Bar = 20 μm. **f**, **g** OIM-C-MSCs were cultured with or without a ROCK inhibitor (Y27632, 50 μM), the non-muscle myosin inhibitor blebbistatin (Blebbist., 50 μM), or an appropriate concentration of DMSO for 5 days. **f** *COL1A1* mRNA expression levels were analyzed by real-time PCR. Data were normalized to the values of C-MSCs treated with DMSO. Values represent means ± S.D. of three cultures (**p < 0.01). **g** Confocal immunofluorescence images show COL1 (green) and nuclei (blue) in C-MSCs. Bar = 100 μm. **h**–**l** C-MSCs transfected with a constitutively active TAZ mutant (TAZS89A) or control plasmid were maintained in GM for 5 days. **h** *COL1A1* mRNA expression levels were analyzed by real-time PCR. Data were normalized to the values of C-MSCs transfected with control vector (cont). Values represent means ± S.D. of three cultures (**p < 0.01). **i** Confocal immunofluorescence images show COL1 (green) and nuclei (blue) in C-MSCs. Bar = 100 μm. **j** Confocal immunofluorescence images of YAP/TAZ/TAZS89A (green), F-actin (red), and nuclei (blue) in C-MSCs. Bar = 20 μm. **k** Immunoblotting for YAP/TAZ in OIM-C-MSCs. A rabbit anti-YAP/TAZ (D24E4) mAb (Cell Signaling) was used to detect YAP, TAZ, and TAZS89A mutant proteins (middle panel). To show YAP expression more clearly, a rabbit anti-YAP (D8H1X) mAb was also used (upper panel). **l** Real-time PCR of *YAP*, *TAZ*, or YAP/TAZ target genes. Data were normalized to the values of C-MSCs transfected with a control vector (cont). Values represent means ± S.D. of three cultures (**p < 0.01)

CTGF, which was upregulated via YAP/TAZ activity in OIM-C-MSCs, can induce COL1 production in MSCs [27]. Accordingly, we hypothesized that upregulated YAP/TAZ activity via OIM treatment was associated with COL1 synthesis, thereby resulting in the activation of a positive feedback mechanotransduction loop in OIM-C-MSCs. Depletion of YAP/TAZ by siRNA co-transfection significantly decreased *COL1A1* mRNA and protein expression levels in OIM-C-MSCs (Fig. 6a, b). Notably, YAP/TAZ siRNA treatment abolished not only YAP/TAZ but also F-actin fibers in OIM-C-MSCs (Fig. 6c). Moreover, Intβ1 siRNA, which reduced F-actin integrity and YAP/TAZ activity in OIM-C-MSCs (Fig. 5a–d), significantly decreased *COL1A1* mRNA and protein expression levels (Fig. 6d, e). Similarly, inhibition of ROCK and non-muscle acto-myosin using Y27632 and blebbistatin, which disrupted F-actin fibers and YAP/TAZ activity in OIM-C-MSCs (Fig. 5f–i), also clearly reduced *COL1* gene and protein expression levels, respectively (Fig. 6f, g). These findings suggested that YAP/TAZ activity caused by OIM treatment can induce COL1 production to activate a positive feedback mechanotransduction loop, which includes Intβ1-ROCK-F-actin formation, in C-MSCs. To support these findings, we examined the effects of constitutively active TAZS89A overexpression on COL1 production, F-actin polymerization, and YAP/TAZ expression levels in C-MSCs, which resulted in decreased YAP/TAZ activity (Fig. 1). TAZS89A-transduced C-MSCs demonstrated increased levels of *COL1A1* mRNA and protein expression (Fig. 6h, i). TAZS89A dramatically stimulated F-actin formation consistent with the elevation of YAP/TAZ/TAZS89A expression and nuclear localization (Fig. 6j). Western blotting analysis demonstrated that transfection of TAZS89A increased not only TAZ/TAZS89A expression but also YAP expression levels in C-MSCs (Fig. 6k). Real-time PCR analysis demonstrated that *TAZ*, *CTGF*, and *CYR61* mRNA expression levels were significantly increased by TAZS89A transfection, although *YAP* mRNA expression levels were not affected (Fig. 6l). These findings suggested that the elevated YAP protein levels in Fig. 6 k were not caused by TAZS89A transcriptional activity but rather by the suppression of proteasomal degradation. Notably, treatment with the actin-disrupting drugs Y27632 and blebbistatin decreased COL1 production and YAP/TAZ activity induced by TAZS89A transfection (Additional file 2: Figure S4). Taken together, these findings showed the presence of a self-reinforcing feed-forward loop between the matrix and YAP/TAZ linked by cytoskeletal mechanisms, similar to the positive feedback regulation described for C-MSCs treated with OIM.

## Discussion

**Fig. 7:**
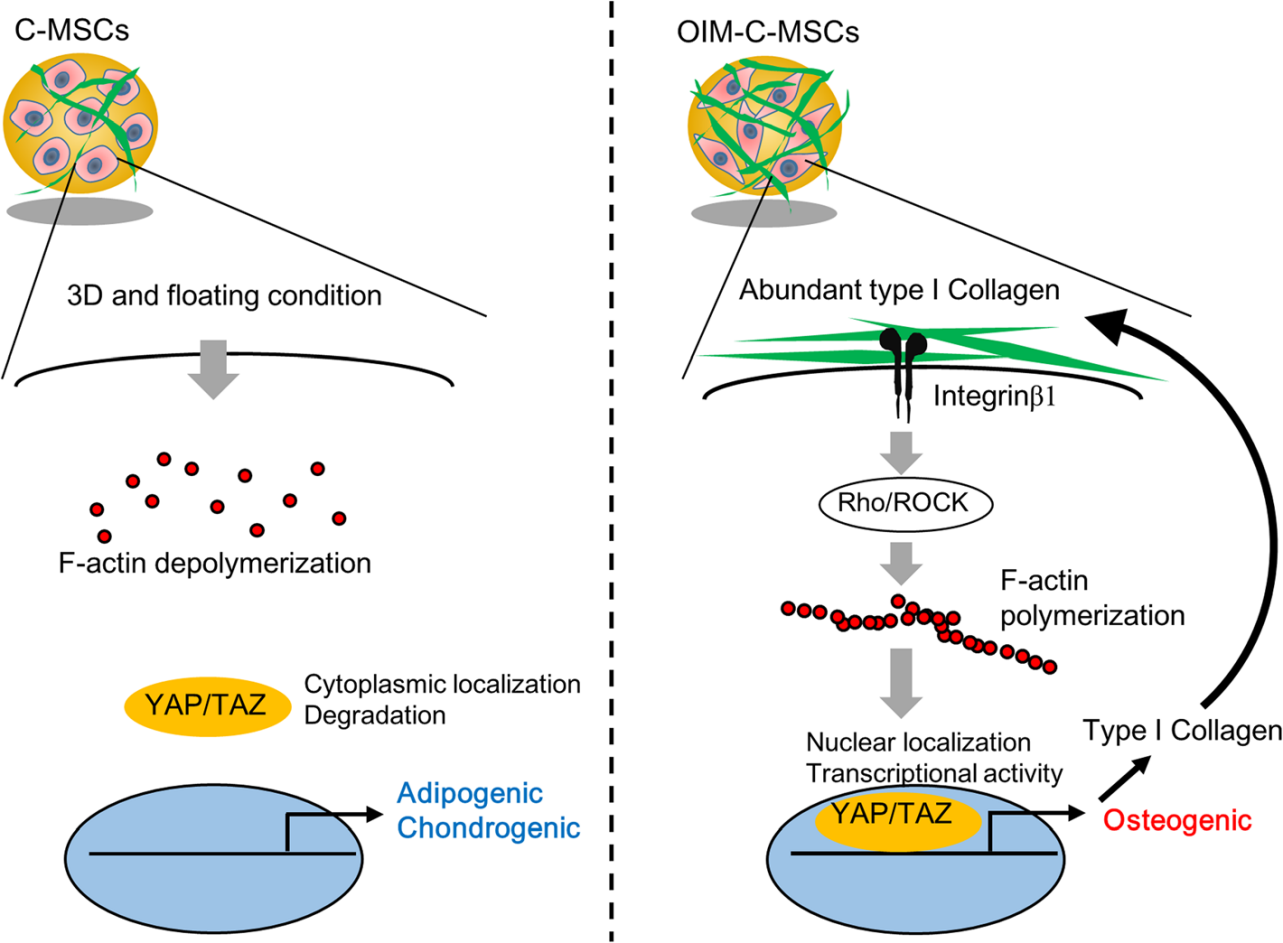
Schematic summary of mechanotransduction cascade in C-MSCs and OIM-C-MSCs

This present study demonstrated that MSCs 2D monolayers detached from the culture dish form 3D structures where YAP/TAZ are inactivated keeping with mechano-regulation of these factors and that culturing floating monolayers in OIM is sufficient to install a feed-forward loop between COL1 production, integrin engagement, acto-myosin contractility, YAP/TAZ activity, which in turn further amplifies COL1 transcription (Fig. 7). These findings indicate for the first time that ECM molecules produced by cell clumps growing in suspension can be sufficient to keep cytoskeletal contractility and YAP/TAZ active. This fact may have broad relevance for the other famous 3D cell construct, such as cell spheroid [28–30] or organoid [31, 32], which are also cultured with suspension condition. Indeed, recent study reported that OIM-pretreated MSC spheroids retain the osteogenic phenotype due to Intα2β1 engagement with the cell-secreted extracellular matrix [33], implying the existence of ECM-mediated YAP/TAZ positive feedback loop. Moreover, Takebe et al. demonstrated that MSC-driven cytoskeletal contraction is indispensable for dynamic formation of various type of organoids [34]. Therefore, this present study may shed light on the key mechano-regulators of 3D cell constructs, which have attracted scientific and medical attention for promising tissue regenerative therapy.

The dominant mechanical cue that occurred during the generation of C-MSCs was the detachment of the cellular sheet from the culture plate. F-actin fibers and YAP/TAZ degradation continued until the end of the C-MSC floating culture period (Fig. 1). In these suspended conditions, it could be challenging to develop sufficient cytoskeletal tension to elevate YAP/TAZ activity in C-MSCs. These findings may suggest that chondrogenic induction of MSCs in vitro preferentially occurs in floating spheroidal culture systems rather than conventional 2D culture on a plastic plate [35]. Because chondrogenesis is abolished by high YAP/TAZ activity [36], the activity in cells attached to a stiff substrate could be too high to induce successful chondrogenesis.

It has been shown that cell detachment from the culture plate or ECM induced a specific type of apoptosis, termed anoikis. Notably, a recent study demonstrated that the loss of attachment by trypsinization reduced cytoskeletal tension to decrease YAP activity, which resulted in the induction of anoikis [23]. Even though this present study also indicated similar mechanotransduction, few apoptotic cells were observed in floating C-MSCs, as described previously [5]. Because C-MSCs consisted of self-produced ECM that could provide the cells with adhesion conditions, compared with single-cell suspension, YAP/TAZ activity in C-MSCs could be maintained at a slightly higher level to escape anoikis.

OIM, which consists of Dex, AA, and Gly, is a frequently employed culture method for tissue engineering approaches or assessment of the osteogenic differentiation capacity of various cell types [37]. Indeed, OIM has been used to demonstrate the relationship between YAP/TAZ signaling and osteogenesis in vitro [22, 38]. However, few studies documenting the direct effects of OIM on YAP/TAZ activity have been reported. This present study demonstrated that OIM stimulated YAP/TAZ activity to increase *RUNX2* mRNA expression in C-MSCs. The key signaling event to induce such YAP/TAZ activity is dependent on an Intβ1-ROCK-F-actin mechanotransduction pathway generated by OIM-induced COL1 deposition. More specifically, we demonstrated that, among the OIM components, the combination of Dex and AA plays a role in COL1 production in association with elevated YAP/TAZ activity in C-MSCs (Fig. 4). Dex upregulates *COL1A1* mRNA expression levels in murine and human MSCs [39–42]. AA increases *COL1* mRNA stability [43] and upregulates secretion of COL1 by inducing proper collagen-triple helix formation [44]. Accordingly, although the precise interaction mechanism of the single elements in OIM is still unclear, Dex and AA may increase COL1 deposition in a coordinated manner to stimulate the YAP/TAZ mechanotransduction pathway in C-MSCs.

The present study revealed that abundant COL1 deposition in OIM-C-MSCs formed a positive feedback loop for YAP/TAZ-regulated mechanotransduction, which was primarily maintained by cytoskeletal tension. In addition, overexpression of TAZS89A also induced a similar feed-forward loop promoting YAP/TAZ activity in C-MSCs. Recently, this type of feed-forward loop of ECM and cytoskeletal tension has been reported in various types of diseases, including cancer [45], lung fibrosis [46], liver fibrosis [47], and pulmonary hypertension [48, 49]. Although aberrant mechanical signaling by YAP/TAZ is associated with the pathogenesis of multiple diseases, YAP/TAZ signaling is also responsible for normal development, organ growth, and tissue regeneration. Therefore, this emerging positive feedback loop of YAP/TAZ may play a role in regulating biological functions in living organisms.

Notably, in contrast to the present study, constitutive active TAZ4SA overexpression in a NIH3T3 mouse fibroblast cell line did not induce ECM deposition or produce a positive feedback loop in other scaffold-free culture systems, i.e., spheroid culture, even though TAZ activity was elevated [50]. The fibroblast spheroids consisted of cells that organize themselves via cell-cell contact. However, no ECM expression was detected in the spheroids by histological analysis. Accordingly, the conflicting findings regarding positive feedback loop formation by transfection of a constitutive active TAZ mutant between C-MSCs and fibroblast spheroids may imply that a certain threshold level of ECM deposition, cytoskeletal tension, and YAP/TAZ activity is essential to generate the feed-forward loop.

Regarding the clinical application of C-MSCs or OIM-C-MSCs for bone regenerative therapy, we reported previously that transplantation of OIM-C-MSCs exerted robust, rapid new bone formation in a bone defect site compared to C-MSCs [4, 6]. This present in vitro study may explain our previous findings. First, C-MSCs, which showed low YAP/TAZ activity, were preferentially induced into adipocytes/chondrocytes but not osteocytes. Second, OIM-C-MSCs showed high YAP/TAZ activity and increased *RUNX2* mRNA expression, which are well-known transcription factors for osteogenic differentiation [51]. Third, OIM-C-MSC transplantation can provide abundant COL1, which has been recognized as an indispensable ECM protein for new bone formation, at a bone defect site [52]. Finally, a positive feedback loop involving COL1 deposition and YAP/TAZ activation may accelerate the bone regenerative properties of OIM-C-MSCs after transplantation. A future study investigating these molecular mechanisms in vivo may provide additional support for novel bone regenerative cell therapies using 3D-cultured C-MSCs.

In this present study, we mainly focused on the cellular property in 5-day OIM-treated C-MSCs. Because the 10-day or 15-day OIM-treated C-MSCs failed to induce bone regeneration in vivo [4, 6]. Interestingly, OIM treatment for more than 10 days induced mineralization, though profound cell apoptosis was also observed in C-MSCs [4, 6]. These findings suggested that excess osteoinduction in vitro may induce C-MSCs to become cellular calcific substances, not biological bone-like tissue. Although the precise molecular mechanism is unclear, mineral deposition may be toxic to the cells in the floating condition which decreased YAP/TAZ activity. To develop the culture protocol that can abrogate the mineral deposition-induced cell apoptosis in floating condition will provide us the biological bone-like tissue in vitro by using C-MSCs.

## Conclusion

This present study clearly demonstrated the distinction of biological capacities between C-MSCs and OIM-C-MSCs in the context of mechanobiology. Briefly, 3D-cultured C-MSCs in floating conditions lost their actin cytoskeleton and had decreased YAP/TAZ activity, which directed cells to undergo adipo-/chondrogenesis. In contrast, OIM treatment facilitated COL1 deposition, which stimulated Intβ1-ROCK-F-actin-YAP/TAZ signaling to induce osteogenesis in C-MSCs. Importantly, the mechanotransduction cascade induced by OIM generated a positive feedback loop of YAP/TAZ activity in C-MSCs. These findings suggest that OIM-C-MSCs, which form a microenvironment that maintains high YAP/TAZ activity, are a superior candidate cell type for bone regenerative cell therapy.

## Additional files

Additional file 1: **Table S1.** Sequences of siRNA oligonucleotides. **Table S2.** Sense and antisense primers for real-time PCR (DOCX 29 kb)

Additional file 2: **Figure S1.** Effect of siRNAs or DNA expression plasmids transfections in C-MSCs. **Figure S2.** MSCs cultured on 2D plastic culture plate maintained YAP/TAZ activity. **Figure S3.** High YAP/TAZ activity in subconfluent MSCs cultured on a 2D plastic plate regulates the cell lineage into osteogenesis but not adipo/chondrogenesis. **Figure S4.** Disruption of F-actin integrity by ROCK inhibitor and acto-myosin inhibitor abrogates the TAZS89A-induced positive feedback loop for in C-MSCs. (ZIP 6647 kb)

## Abbreviations

2D/3D: 2/3-Dimensional
AIM: Adipogenic induction medium
AP-2: Activating protein 2
CIM: Chondrogenic induction medium
C-MSCs: Clumps of mesenchymal stem cells/extracellular matrix proteins
COL1: Collagen type I
CTGF: Connective tissue growth factor
CYR61: Cysteine-rich angiogenic inducer 61
DAPI: 4′6-Diamidino-2-phenylindole
DMEM: Dulbecco’s modified Eagle’s medium
DMSO: Dimethyl sulfoxide
ECM: Extracellular matrix
F-actin: Filamentous actin
FBS: Fetal bovine serum
Intβ1: Integrinβ1
MSCs: Mesenchymal stem cells
OCN: Osteocalcin
OIM: Osteogenic induction medium
PPARγ: Peroxisome proliferator-activated receptor γ
ROCK: Rho-associated protein kinase
RUNX2: Runt-related transcription factor 2
Sox9: SRY (sex determining region Y)-box 9
TAZ: Transcriptional co-activator with PDZ-binding motif
TEAD: TEA transcriptional factor
YAP: Yes-associated protein
