# Supplementary material for: Type I collagen deposition via osteoinduction ameliorates YAP/TAZ activity in 3D floating culture clumps of mesenchymal stem cell/extracellular matrix complexes
Source: Stem Cell Res Ther. 2018 Dec 7;9:342. doi: 10.1186/s13287-018-1085-9 (PMC6286508; doi:10.1186/s13287-018-1085-9)
Supplement: Supplementary file 1 — Table S1. Sequences of siRNA oligonucleotides. Table S2. Sense and antisense primers for real-time PCR (DOCX 29 kb) [file 13287_2018_1085_MOESM1_ESM.docx]

**Table S1. Sequences of siRNA oligonucleotides**

| *siRNA* | *Sense strand sequence* |
| --- | --- |
| YAP | GACAUCUUCUGGUCAGAGA dTdT |
|  |  |
| TAZ | ACGUUGACUUAGGAACUUU dTdT |
|  |  |
| Control | UUCUCCGAACGUGUCACGU dTdT |

**Table S2. Sense and antisense primers for real-time PCR**

| *Target gene* |  |  | *Primer sequence* |  |  |
| --- | --- | --- | --- | --- | --- |
| CTGF | Forward | | 5'- AGGAGTGGGTGTGTGACGA -3' | | |
|  | Reverse | | 5'- CCAGGCAGTTGGCTCTAATC -3' | | |
|  |  | |  | | |
| CYR61 | Forward | | 5'- CCTTGTGGACAGCCAGTGTA -3' | | |
|  | Reverse | | 5'- ACTTGGGCCGGTATTTCTTC -3' | | |
|  |  | |  | | |
| YAP | Forward | | 5'- GATCCCTGATGATGTACCACTGCC -3' | | |
|  | Reverse | | 5'- GCCATGTTGTTGTCTGATCGTTGTG -3' | | |
|  |  | |  | | |
| TAZ | Forward | | 5'- GTCACCAACAGTAGCTCAGATC -3' | | |
|  | Reverse | | 5'- AGTGATTACAGCCAGGTTAGAAAG -3' | | |
|  |  | |  | | |
| RUNX2 | Forward | | 5'- ACTACCAGCCACCGAGACCA -3' | | |
|  | Reverse | | 5'- ACTGCTTGCAGCCTTAAATGACTC -3' | | |
|  |  | |  | | |
| OCN | Forward | | 5'- GTGACGAGTTGGCTGACC -3' | | |
|  | Reverse | | 5'- TGGAGAGGAGCAGAACTGG -3' | | |
|  |  | |  | | |
| COL1A1 | Forward | | 5'- GCTGGTCACCATGGTGATCAAGG -3' | | |
|  | Reverse | | 5'- TACCAGGATGTCCAGTGCGAC -3' | | |
|  |  | |  | | |
| PPARγ | Forward | | 5'- CTTGCAGTGGGGATGT-3' | | |
|  | Reverse | | 5'- CTTTGGTCAGCGGGAA -3' | | |
|  |  | |  | | |
| AP2 | Forward | | 5'- ATTTGACGAAGTCACTGC -3' | | |
|  | Reverse | | 5'- CATCCTCTCGTTTTCTCTTTAT -3' | | |
|  |  | |  | | |
| SOX9 | Forward | | 5'-CATGAGCGAGGTGCACTCC-3' | | |
|  | Reverse | | 5'- TCGCTTCAGGTCAGCCTTG-3' | | |
|  |  | |  | | |
| Aggrecan | Forward | | 5'- TGCATTCCACGAAGCTAACCTT -3' | | |
|  | Reverse | | 5'- GACGCCTCGCCTTCTTGAA -3' | | |
|  |  | |  | | |
| 18S | Forward | | 5'- GTAACCCGTTGAACCCCATT -3' | | |
|  | Reverse | | 5'- CCATCCAATCGGTAGTAGCG -3' | | |
