# Supplementary material for: Type I collagen deposition via osteoinduction ameliorates YAP/TAZ activity in 3D floating culture clumps of mesenchymal stem cell/extracellular matrix complexes
Source: Stem Cell Res Ther. 2018 Dec 7;9:342. doi: 10.1186/s13287-018-1085-9 (PMC6286508; doi:10.1186/s13287-018-1085-9)
Supplement: Supplementary file 2 — Figure S1. Effect of siRNAs or DNA expression plasmids transfections in C-MSCs. Figure S2. MSCs cultured on 2D plastic culture plate maintained YAP/TAZ activity. Figure S3. High YAP/TAZ activity in subconfluent MSCs cultured on a 2D plastic plate regulates the cell lineage into osteogenesis but not adipo/chondrogenesis. Figure S4. Disruption of F-actin integrity by ROCK inhibitor and acto-myosin inhibitor abrogates the TAZS89A-induced positive feedback loop for in C-MSCs. (ZIP 6647 kb) [file 13287_2018_1085_MOESM2_ESM.zip › Supplemental figure legends for SCRT.docx]

**Additional file figure legends**

*Figure S1. Effect of siRNAs or DNA expression plasmids transfections in C-MSCs.*

(A and D) Schematic images of siRNA (A) and DNA plasmid (D) transfection procedures in C-MSCs. See details in the Experimental procedures section. (B and C) Immunoblotting for YAP and TAZ (B), or Intβ1 (C) in cells transfected with each specific siRNA. (E) Immunoblotting for YAP/TAZ in cells transfected with control or TAZS89A expression plasmids. A rabbit anti-YAP/TAZ (D24E4) mAb (Cell Signaling) was employed to detect YAP, TAZ, and TAZS89A mutant proteins.

*Figure S2. MSCs cultured on 2D plastic culture plate maintained YAP/TAZ activity.*

(A). Schematic image of 2D MSCs culture. To clarify the effect of 3D culture in Figure 1 on YAP/TAZ activity, some MSCs were maintained on 2D culture plates without detachment. Then, the cells were cultured in GM for the indicated period. (B) Confocal immunofluorescence images of YAP/TAZ (green), F-actin (red), and nuclei (blue) in MSCs. Bar = 20 μm. (C) The graph summarizes the distribution of YAP/TAZ patterns. The patterns were classified as mainly nuclear (N>C), diffuse (N=C), and mainly cytoplasmic (N<C). (D) Immunoblotting for YAP/TAZ in MSCs. (E) Real-time PCR for YAP/TAZ target genes in MSCs. Data are normalized to day 0. Values represent means ± S.D. of three cultures (** *p* < 0.01). All graphs and images are representative of three independent experiments.

*Figure S3. High YAP/TAZ activity in subconfluent MSCs cultured on a 2D plastic plate regulates the cell lineage into osteogenesis but not adipo/chondrogenesis.*

(A-F) MSCs were seeded at a density of 5.0 × 10^3^ cells/well into 6-well culture plates and maintained with GM for 3 days. Then, the cells were cultured in (C) OIM, (D) AIM, (E) CIM or (F) Dual medium for 5 days. (A) Before induction, the cells were fixed and the expression patterns of YAP/TAZ (green), F-actin (red), and nuclei (blue) were analyzed using confocal immunofluorescence microscopy. Bar = 20 μm. (B) The graph summarizes the distribution of YAP/TAZ patterns. The patterns were classified as mainly nuclear (N>C), diffuse (N=C), and mainly cytoplasmic (N<C). (C-F) Differentiation marker gene expression levels were analyzed by real-time PCR. Data were normalized to MSCs maintained with GM. Values represent means ± S.D. of three cultures (** *p* < 0.01). (G-J) MSCs were seeded at a density of 5.0 × 10^3^ cells/well into 6-well culture plates and maintained with GM for 2 days. Then, YAP and TAZ siRNAs were co-transfected by lipofection. After a 1-day incubation, some cells were isolated for immunoblotting (G) and others were cultured in (H) OIM, (I) AIM, or (J) CIM for 5 days,. (G) Immunoblotting for YAP/TAZ in MSCs transfected with siRNAs. (H-J) Differentiation marker gene expression levels were analyzed by real-time PCR. Data were normalized to MSCs transfected with control siRNA (sicont). Values represent means ± S.D. of three cultures (** *p* < 0.01). All graphs and images are representative of three independent experiments.

*Figure S4. Disruption of F-actin integrity by ROCK inhibitor and acto-myosin inhibitor abrogates the TAZS89A-induced positive feedback loop for in C-MSCs.*

(A-D) C-MSCs transfected with a TAZ constitutively active mutant (TAZS89A) were maintained in GM with or without ROCK inhibitor (Y27632, 50 μM), non-muscle myosin inhibitor blebbistatin (Blebbist., 50 μM), or appropriate concentrations of DMSO for 5 days. (A) Confocal immunofluorescence images show COL1 (green), and nuclei (blue) in C-MSCs. Bar = 100 μm. (B) Confocal immunofluorescence images of YAP/TAZ/TAZS89A (green), F-actin (red), and nuclei (blue) in C-MSCs. Bar = 20 μm. (C) The graph summarizes the distribution of YAP/TAZ/TAZS89A localizing patterns. The patterns were classified as mainly nuclear (N>C), diffuse (N=C), and mainly cytoplasmic or undetectable (N<C or undetectable). (D) Immunoblotting for YAP/TAZ in C-MSCs. A rabbit anti-YAP/TAZ (D24E4) mAb (Cell Signaling) was employed to detect YAP, TAZ, and TAZS89A mutant proteins (middle panel). To show YAP expression more clearly, a rabbit anti-YAP (D8H1X) mAb was also tested (upper panel). All graphs and images are representative of three independent experiments.
