# Supplementary figures and images for: Type I collagen deposition via osteoinduction ameliorates YAP/TAZ activity in 3D floating culture clumps of mesenchymal stem cell/extracellular matrix complexes
Source: Stem Cell Res Ther. 2018 Dec 7;9:342. doi: 10.1186/s13287-018-1085-9 (PMC6286508; doi:10.1186/s13287-018-1085-9)

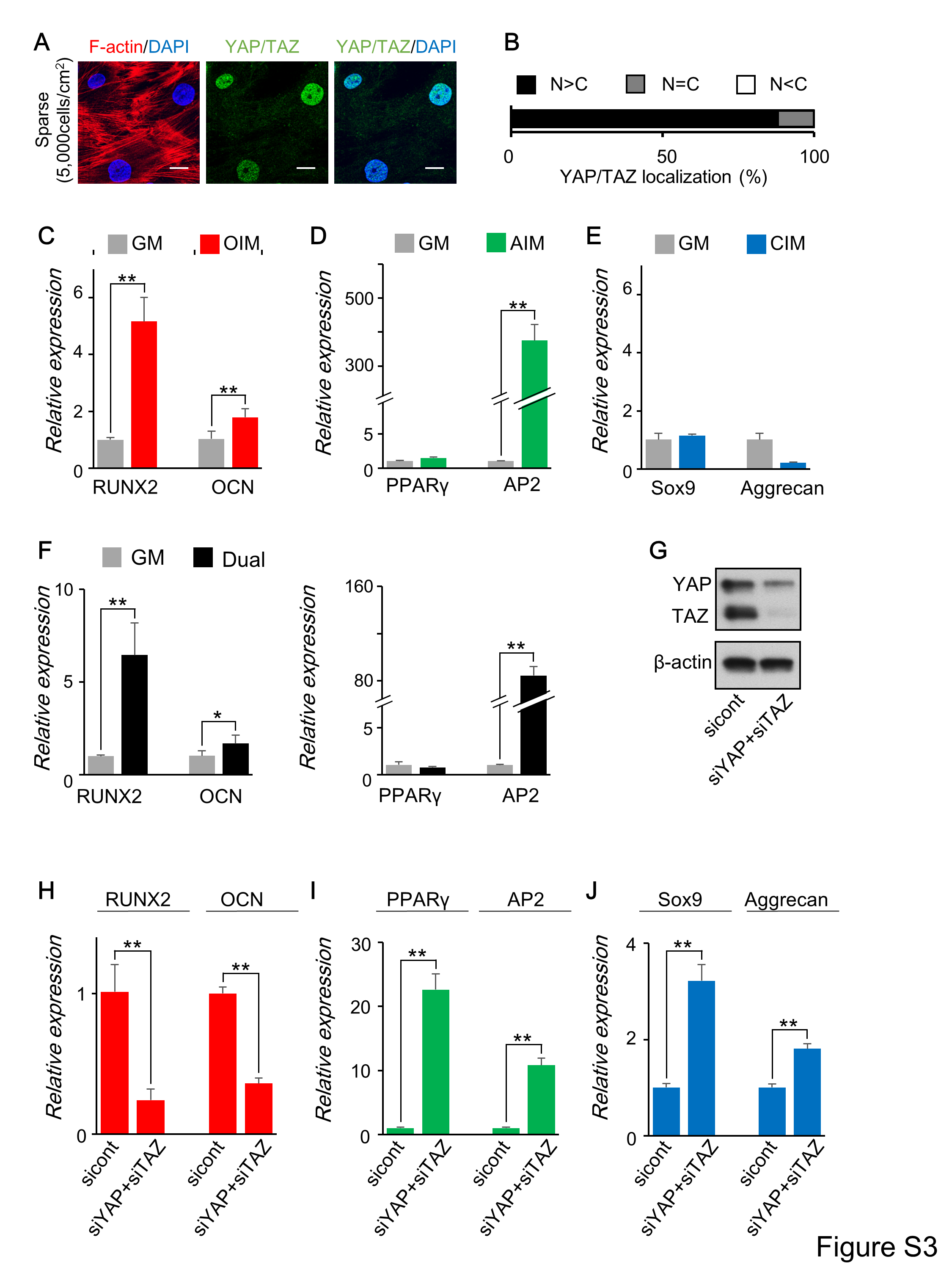

Supplement: Supplementary file 2 — Figure S1. Effect of siRNAs or DNA expression plasmids transfections in C-MSCs. Figure S2. MSCs cultured on 2D plastic culture plate maintained YAP/TAZ activity. Figure S3. High YAP/TAZ activity in subconfluent MSCs cultured on a 2D plastic plate regulates the cell lineage into osteogenesis but not adipo/chondrogenesis. Figure S4. Disruption of F-actin integrity by ROCK inhibitor and acto-myosin inhibitor abrogates the TAZS89A-induced positive feedback loop for in C-MSCs. (ZIP 6647 kb) [file 13287_2018_1085_MOESM2_ESM.zip › supplemental Fig. 3.TIF]

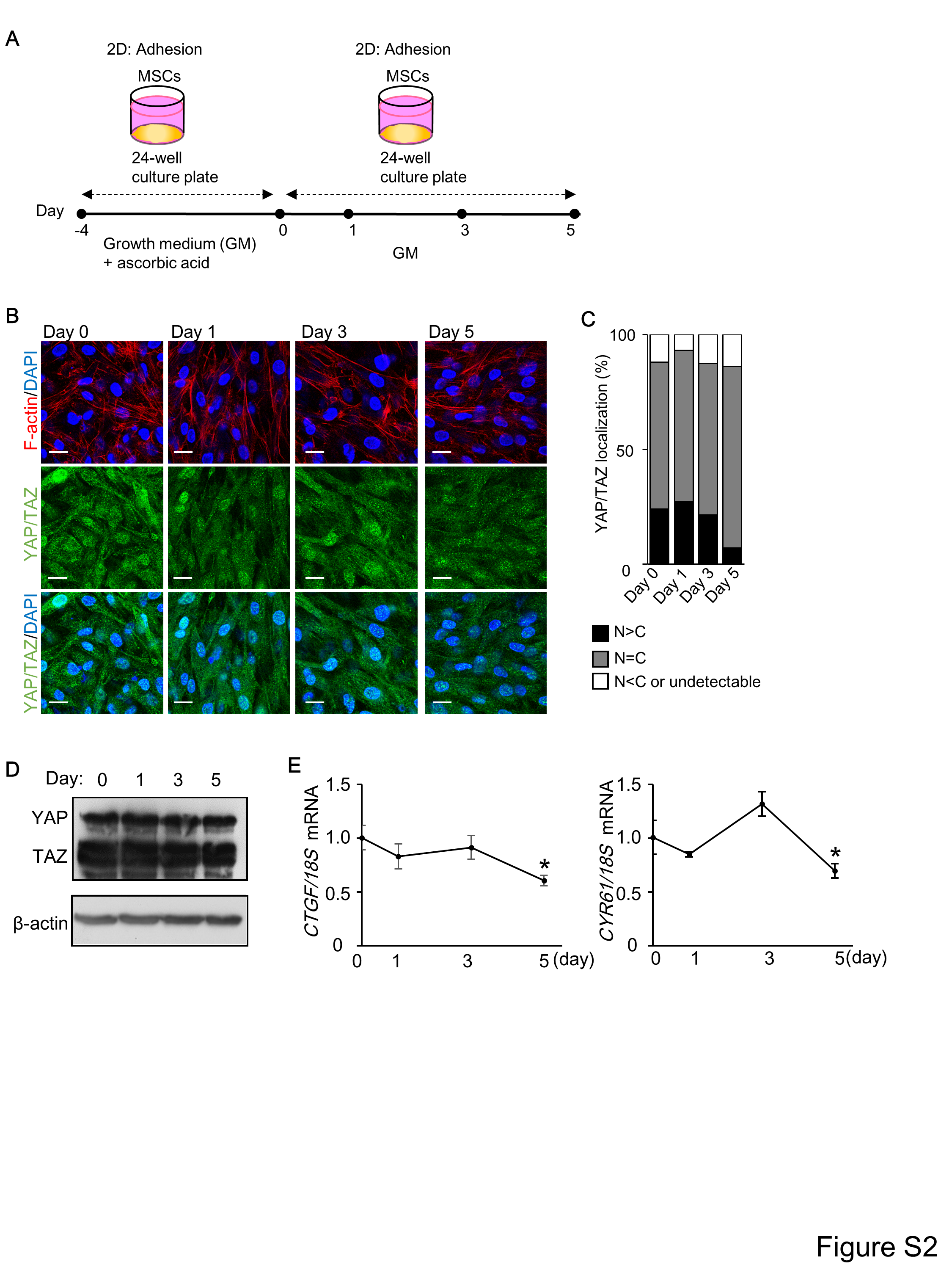

Supplement: Supplementary file 2 — Figure S1. Effect of siRNAs or DNA expression plasmids transfections in C-MSCs. Figure S2. MSCs cultured on 2D plastic culture plate maintained YAP/TAZ activity. Figure S3. High YAP/TAZ activity in subconfluent MSCs cultured on a 2D plastic plate regulates the cell lineage into osteogenesis but not adipo/chondrogenesis. Figure S4. Disruption of F-actin integrity by ROCK inhibitor and acto-myosin inhibitor abrogates the TAZS89A-induced positive feedback loop for in C-MSCs. (ZIP 6647 kb) [file 13287_2018_1085_MOESM2_ESM.zip › supplemental Fig. 2.TIF]

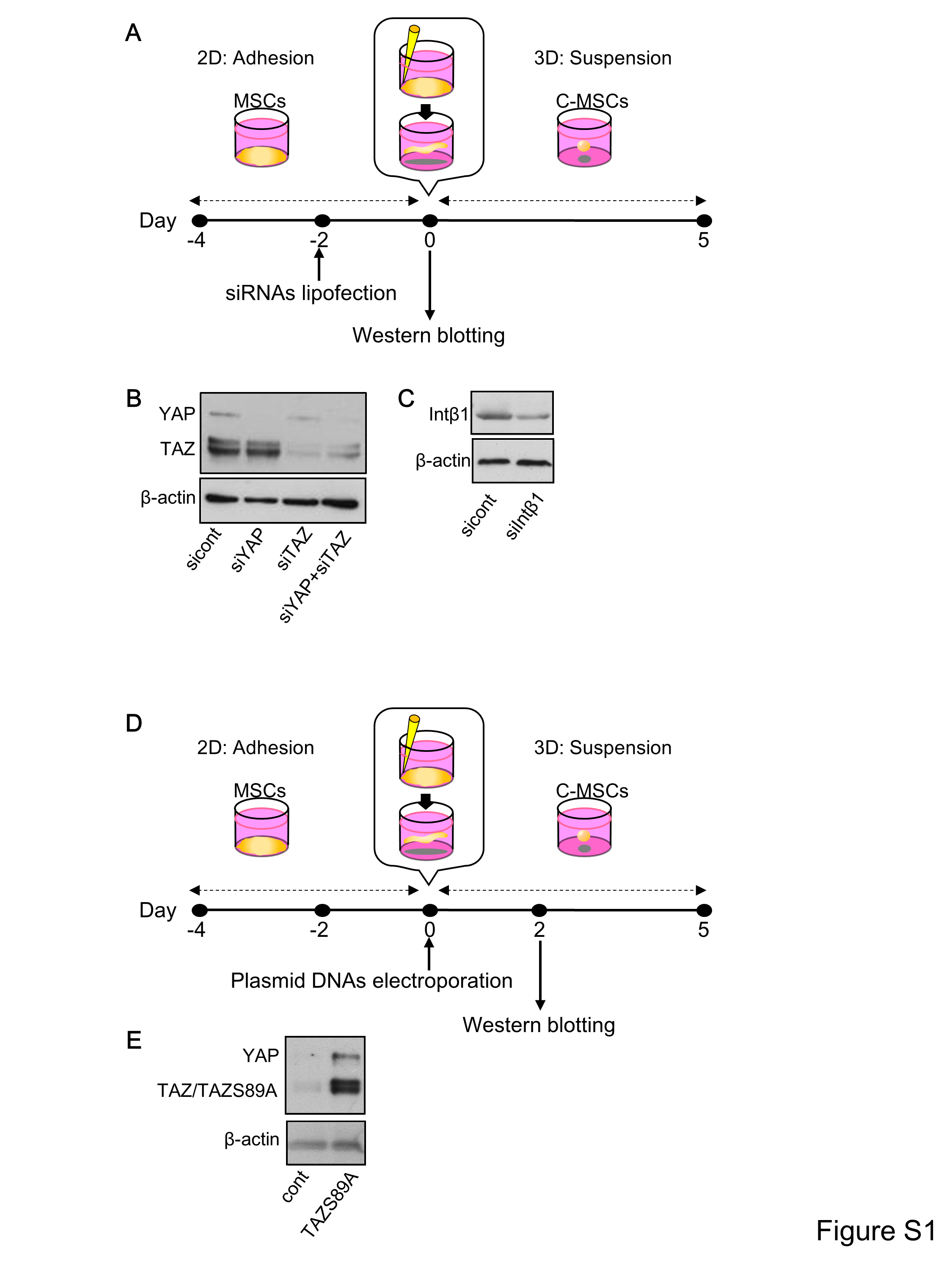

Supplement: Supplementary file 2 — Figure S1. Effect of siRNAs or DNA expression plasmids transfections in C-MSCs. Figure S2. MSCs cultured on 2D plastic culture plate maintained YAP/TAZ activity. Figure S3. High YAP/TAZ activity in subconfluent MSCs cultured on a 2D plastic plate regulates the cell lineage into osteogenesis but not adipo/chondrogenesis. Figure S4. Disruption of F-actin integrity by ROCK inhibitor and acto-myosin inhibitor abrogates the TAZS89A-induced positive feedback loop for in C-MSCs. (ZIP 6647 kb) [file 13287_2018_1085_MOESM2_ESM.zip › supplemental Fig. 1.TIF]

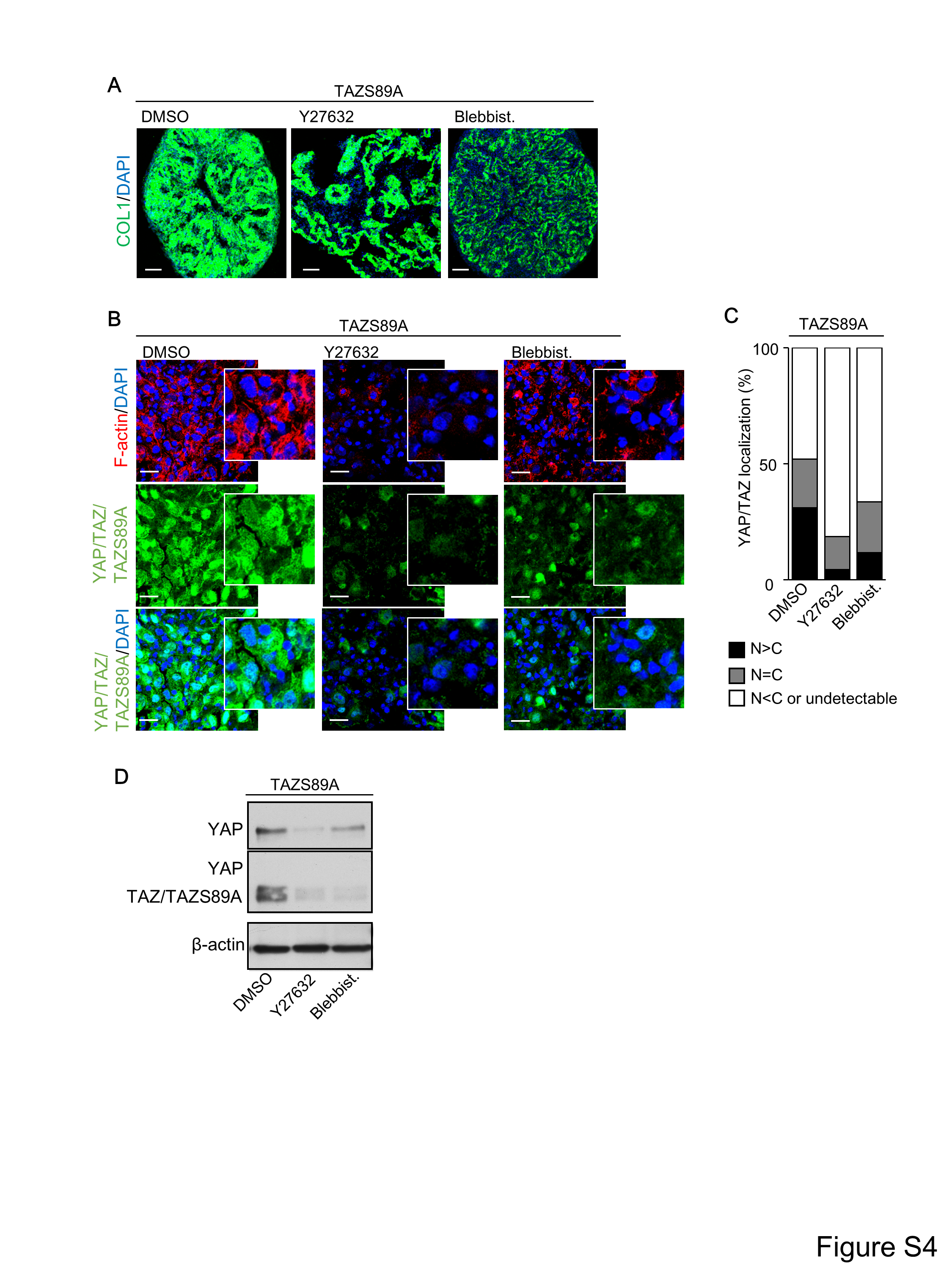

Supplement: Supplementary file 2 — Figure S1. Effect of siRNAs or DNA expression plasmids transfections in C-MSCs. Figure S2. MSCs cultured on 2D plastic culture plate maintained YAP/TAZ activity. Figure S3. High YAP/TAZ activity in subconfluent MSCs cultured on a 2D plastic plate regulates the cell lineage into osteogenesis but not adipo/chondrogenesis. Figure S4. Disruption of F-actin integrity by ROCK inhibitor and acto-myosin inhibitor abrogates the TAZS89A-induced positive feedback loop for in C-MSCs. (ZIP 6647 kb) [file 13287_2018_1085_MOESM2_ESM.zip › revised supply Fig. 4.tif]
